# Diaqua­bis(2,4-dichloro-6-formyl­phenolato)zinc(II)–bis­(μ-2,4-dichloro-6-formyl­phenolato)bis­[aqua­(2,4-dichloro-6-formyl­phenolato)zinc(II)] (2/1)

**DOI:** 10.1107/S160053680904896X

**Published:** 2009-11-21

**Authors:** Yoshimasa Watanabe, Yoshikazu Aritake, Takashiro Akitsu

**Affiliations:** a Department of Chemistry, Faculty of Science, Tokyo University of Science, 1-3 Kagurazaka, Shinjuku-ku, Tokyo 162-8601, Japan

## Abstract

The crystal of the title compound, [Zn(C_7_H_3_Cl_2_O_2_)_2_(H_2_O)_2_]_2_·[Zn_2_(C_7_H_3_Cl_2_O_2_)_4_(H_2_O)_2_], consists of monomeric and dimeric Zn^II^ complexes. Both complexes afford a six-coordinated coordination environment about the Zn atoms with *cis*-configuration ligands. The deprotonated hydr­oxy groups of the 3,5-dichloro­salicylaldehyde ligands bridge two metal cations, forming a centrosymmetric dimeric complex. Inter­molecular O—H⋯O hydrogen bonding occurs between the coordinated water mol­ecules and deprotonated hydr­oxy groups in the crystal structure.

## Related literature

For applications of the 3,5-dichlorosalicylaldehydate ligand in the preparation of Schiff base–metal complexes, see: Akitsu *et al.* (2009 ▶); Akitsu & Einaga (2005*a* ▶,*b* ▶); Akitsu (2007 ▶). For *trans* and *cis* forms of complexes, see: Akitsu & Einaga (2004*a* ▶,*b* ▶); Akitsu *et al.* (2005 ▶). For related complexes, see: Chen (2006 ▶); Chen *et al.* (2007 ▶); Xiong & Liu (2005 ▶).
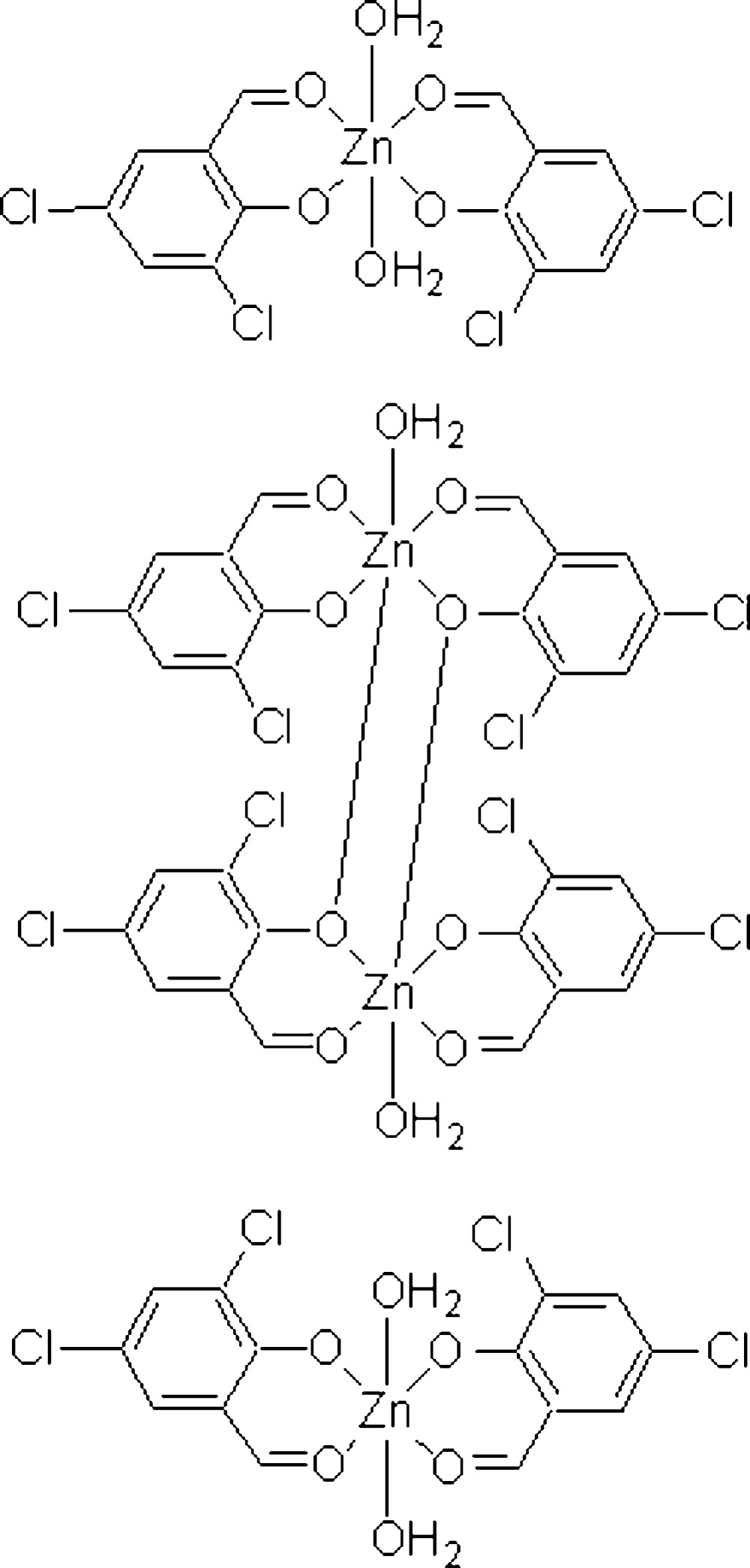


## Experimental

### 

#### Crystal data

[Zn(C_7_H_3_Cl_2_O_2_)_2_(H_2_O)_2_]_2_·[Zn_2_(C_7_H_3_Cl_2_O_2_)_4_(H_2_O)_2_]
*M* *_r_* = 1889.61
Triclinic, 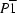

*a* = 8.7532 (9) Å
*b* = 13.6973 (15) Å
*c* = 14.2833 (15) Å
α = 96.244 (2)°
β = 91.700 (1)°
γ = 106.096 (1)°
*V* = 1632.4 (3) Å^3^
*Z* = 1
Mo *K*α radiation
μ = 2.19 mm^−1^
*T* = 100 K
0.15 × 0.15 × 0.08 mm

#### Data collection

Bruker APEXII CCD area-detector diffractometer
Absorption correction: multi-scan (*SADABS*; Sheldrick, 1996 ▶) *T*_min_ = 0.735, *T*_max_ = 0.845
9504 measured reflections
7275 independent reflections
5671 reflections with *I* > 2σ(*I*)
*R*_int_ = 0.021

#### Refinement

*R*[*F* ^2^ > 2σ(*F* ^2^)] = 0.038
*wR*(*F* ^2^) = 0.118
*S* = 0.75
7275 reflections
445 parameters
H-atom parameters constrained
Δρ_max_ = 0.59 e Å^−3^
Δρ_min_ = −0.46 e Å^−3^

### 

Data collection: *APEX2* (Bruker, 2005 ▶); cell refinement: *SAINT* (Bruker, 2005 ▶); data reduction: *SAINT*; program(s) used to solve structure: *SHELXS97* (Sheldrick, 2008 ▶); program(s) used to refine structure: *SHELXL97* (Sheldrick, 2008 ▶); molecular graphics: *ORTEP-3 for Windows* (Farrugia, 1997 ▶); software used to prepare material for publication: *SHELXL97*.

## Supplementary Material

Crystal structure: contains datablocks global, I. DOI: 10.1107/S160053680904896X/xu2645sup1.cif

Structure factors: contains datablocks I. DOI: 10.1107/S160053680904896X/xu2645Isup2.hkl

Additional supplementary materials:  crystallographic information; 3D view; checkCIF report

## Figures and Tables

**Table 1:** Selected bond lengths (Å)

Zn1—O1	2.040 (2)
Zn1—O2	2.096 (2)
Zn1—O3	2.049 (2)
Zn1—O4	2.084 (2)
Zn1—O9	2.112 (2)
Zn1—O10	2.130 (2)
Zn2—O5	2.011 (2)
Zn2—O6	2.114 (2)
Zn2—O7	2.081 (2)
Zn2—O7^i^	2.176 (2)
Zn2—O8	2.069 (2)
Zn2—O11	2.134 (2)

Symmetry code: (i) 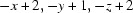.

**Table 2:** Hydrogen-bond geometry (Å, °)

*D*—H⋯*A*	*D*—H	H⋯*A*	*D*⋯*A*	*D*—H⋯*A*
O9—H9*A*⋯O3^ii^	0.84	2.02	2.790 (3)	153
O9—H9*B*⋯O1^ii^	0.74	2.45	3.015 (3)	134
O10—H10*A*⋯O7^i^	0.84	2.24	2.998 (3)	150
O10—H10*B*⋯O5^i^	0.82	1.95	2.741 (3)	161
O11—H11*A*⋯O3^i^	0.84	2.17	2.931 (3)	151
O11—H11*B*⋯O1^i^	0.84	1.93	2.751 (3)	169

Symmetry codes: (i) 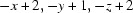; (ii) 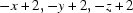.

**Fig. 1.**
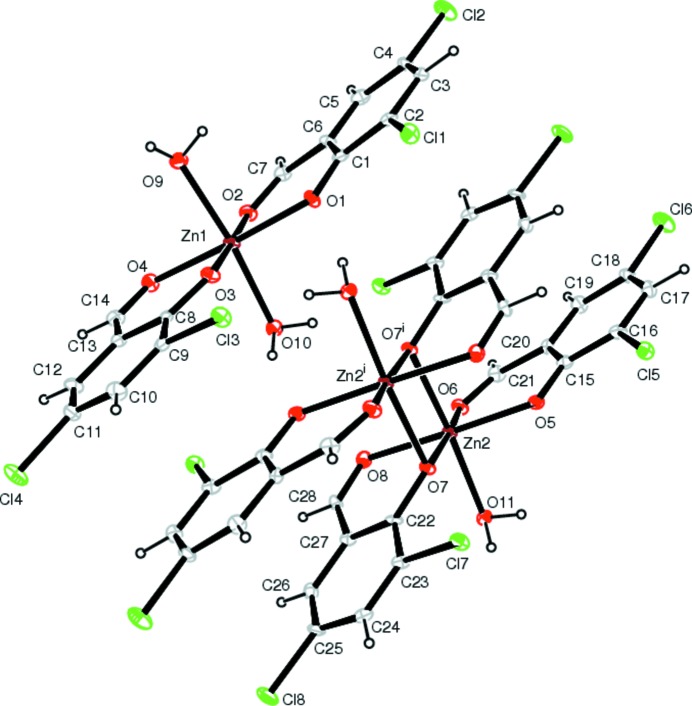
The molecular structure of (I), showing the atom labeling scheme [symmetry code: (i) 2 - x, 1 - y, 2 - z]. Displacement ellipsoids for non-H atoms are drawn at the 50% probability level.

## supplementary crystallographic information

### Comment

3,5-Dichlorosalcylaldehydato plays an important role in preparation of Schiff base metal complexes because of electronic properties due to Cl-groups for example supramolecular interactions between metallodendrimers (Akitsu *et al.*, 2009) or photochromic compounds (Akitsu & Einaga, 2005*a* & 2005*b*; Akitsu, 2007). Depending on amine reagents and their steric requirement, *trans* (Akitsu & Einaga, 2004*a* & 2004*b*) or *cis* (Akitsu *et al.*, 2005) forms of complexes can be formed. However, we focused on only 3,5-dichloroaldehyde moiety to elucidate structural features without amine moiety.

The title compound (I) is composed of a co-crystal of monomeric [Zn(C_7_H_3_Cl_2_O_2_)_2_(H_2_O)_2_] and dimeric [Zn(C_7_H_3_Cl_2_O_2_)_2_(H_2_O)]. Both complexes afford a six-coordinated coordination environment exhibiting significant distortion. In contrast to known zinc(II) complexes incorporating saldehyde-derivertive ligands (Chen, 2006; Chen *et al.*, 2007; Xiong & Liu, 2005), both ligands bind to Zn(II) ions in a *cis*-configuration for (I).

### Experimental

Crystals were obtained accidentally as a byproduct of the treatment of 3,5-dichlorosalcylaldehyde (0.95 g, 5.00 mmol) in methanol (30 ml), *L*-alanine (0.44 g, 5.00 mmol) in water (5 ml), zinc(II) acetate dihydrate (0.55 g, 2.50 mmol) and several drops of triethylamine at c.a. 350 K for 2 hr.

### Refinement

Water-H atoms were located based on D-map and refined in riding mode. Other H atoms were placed at the calculated positions with C—H = 0.95 Å and refined in riding mode. U_iso_(H) = 1.2U_eq_(O,C).

### Crystal data

**Table:**

[Zn(C_7_H_3_Cl_2_O_2_)_2_(H_2_O)_2_]_2_·[Zn_2_(C_7_H_3_Cl_2_O_2_)_4_(H_2_O)_2_]	*Z* = 1
*M**_r_* = 1889.61	*F*(000) = 940.0
Triclinic, *P*1	*D*_x_ = 1.922 Mg m^−^^3^
Hall symbol: -P 1	Mo *K*α radiation, λ = 0.71073 Å
*a* = 8.7532 (9) Å	Cell parameters from 2792 reflections
*b* = 13.6973 (15) Å	θ = 2.4–27.8°
*c* = 14.2833 (15) Å	µ = 2.19 mm^−^^1^
α = 96.244 (2)°	*T* = 100 K
β = 91.700 (1)°	Prismatic, yellow
γ = 106.096 (1)°	0.15 × 0.15 × 0.08 mm
*V* = 1632.4 (3) Å^3^	

### Data collection

**Table:**

Bruker APEXII CCD area-detector diffractometer	7275 independent reflections
Radiation source: fine-focus sealed tube	5671 reflections with *I* > 2σ(*I*)
graphite	*R*_int_ = 0.021
Detector resolution: 8.333 pixels mm^-1^	θ_max_ = 27.9°, θ_min_ = 1.4°
φ and ω scans	*h* = −11→11
Absorption correction: multi-scan (*SADABS*; Sheldrick, 1996)	*k* = −16→17
*T*_min_ = 0.735, *T*_max_ = 0.845	*l* = −12→18
9504 measured reflections	

### Refinement

**Table:**

Refinement on *F*^2^	Primary atom site location: structure-invariant direct methods
Least-squares matrix: full	Secondary atom site location: difference Fourier map
*R*[*F*^2^ > 2σ(*F*^2^)] = 0.038	Hydrogen site location: inferred from neighbouring sites
*wR*(*F*^2^) = 0.118	H-atom parameters constrained
*S* = 0.75	*w* = 1/[σ^2^(*F*_o_^2^) + (0.1*P*)^2^ + 1.2111*P*] where *P* = (*F*_o_^2^ + 2*F*_c_^2^)/3
7275 reflections	(Δ/σ)_max_ = 0.001
445 parameters	Δρ_max_ = 0.59 e Å^−^^3^
0 restraints	Δρ_min_ = −0.46 e Å^−^^3^

### Special details

**Table:**

Experimental. Refinement of F^2^ against ALL reflections. The weighted *R*-factor *wR* and goodness of fit S are based on F^2^, conventional *R*-factors *R* are based on F, with F set to zero for negative F^2^. The threshold expression of F^2^ > 2sigma(F^2^) is used only for calculating *R*-factors(gt) *etc*. and is not relevant to the choice of reflections for refinement. *R*-factors based on F^2^ are statistically about twice as large as those based on F, and R– factors based on ALL data will be even larger. The water-H atoms wre located in a D-map and refined in riding mode with *U*_iso_(H) = 1.2*U*_eq_(O)."
Geometry. All e.s.d.'s (except the e.s.d. in the dihedral angle between two l.s. planes) are estimated using the full covariance matrix. The cell e.s.d.'s are taken into account individually in the estimation of e.s.d.'s in distances, angles and torsion angles; correlations between e.s.d.'s in cell parameters are only used when they are defined by crystal symmetry. An approximate (isotropic) treatment of cell e.s.d.'s is used for estimating e.s.d.'s involving l.s. planes.

### Fractional atomic coordinates and isotropic or equivalent isotropic displacement parameters (Å^2^)

**Table:**

	*x*	*y*	*z*	*U*_iso_*/*U*_eq_	
Zn1	0.80461 (4)	0.83299 (3)	0.96067 (3)	0.00981 (11)	
Zn2	0.92258 (4)	0.39423 (3)	0.93218 (3)	0.00861 (10)	
C1	1.0506 (4)	0.8558 (2)	0.8153 (2)	0.0115 (7)	
C2	1.2112 (4)	0.8851 (2)	0.7897 (2)	0.0106 (7)	
C3	1.2547 (4)	0.8968 (2)	0.6987 (2)	0.0114 (7)	
H3	1.3640	0.9165	0.6855	0.014*	
C4	1.1363 (4)	0.8794 (3)	0.6263 (2)	0.0123 (7)	
C5	0.9784 (4)	0.8516 (3)	0.6459 (2)	0.0144 (7)	
H5	0.8984	0.8400	0.5963	0.017*	
C6	0.9340 (4)	0.8400 (2)	0.7388 (2)	0.0109 (7)	
C7	0.7636 (4)	0.8084 (2)	0.7507 (2)	0.0122 (7)	
H7	0.6966	0.7972	0.6946	0.015*	
C8	0.8264 (4)	0.8649 (2)	1.1745 (2)	0.0089 (6)	
C9	0.9119 (4)	0.8798 (2)	1.2634 (2)	0.0111 (7)	
C10	0.8403 (4)	0.8716 (3)	1.3478 (2)	0.0152 (7)	
H10C	0.9029	0.8815	1.4053	0.018*	
C11	0.6749 (4)	0.8485 (3)	1.3479 (2)	0.0140 (7)	
C12	0.5844 (4)	0.8358 (3)	1.2644 (2)	0.0134 (7)	
H12	0.4719	0.8215	1.2653	0.016*	
C13	0.6573 (4)	0.8437 (2)	1.1784 (2)	0.0105 (6)	
C14	0.5495 (4)	0.8274 (2)	1.0955 (2)	0.0130 (7)	
H14	0.4403	0.8178	1.1070	0.016*	
C15	1.1453 (4)	0.3785 (2)	0.7795 (2)	0.0105 (6)	
C16	1.3048 (4)	0.3981 (2)	0.7511 (2)	0.0107 (7)	
C17	1.3436 (4)	0.4026 (2)	0.6585 (2)	0.0119 (7)	
H17	1.4517	0.4171	0.6429	0.014*	
C18	1.2213 (4)	0.3855 (3)	0.5875 (2)	0.0127 (7)	
C19	1.0649 (4)	0.3623 (3)	0.6098 (2)	0.0133 (7)	
H19	0.9827	0.3478	0.5611	0.016*	
C20	1.0254 (4)	0.3598 (2)	0.7050 (2)	0.0109 (6)	
C21	0.8572 (4)	0.3365 (3)	0.7224 (2)	0.0130 (7)	
H21	0.7847	0.3167	0.6683	0.016*	
C22	0.9237 (4)	0.4081 (2)	1.1431 (2)	0.0089 (6)	
C23	0.9985 (4)	0.4002 (2)	1.2300 (2)	0.0103 (6)	
C24	0.9167 (4)	0.3743 (2)	1.3089 (2)	0.0125 (7)	
H24	0.9724	0.3710	1.3660	0.015*	
C25	0.7501 (4)	0.3529 (3)	1.3036 (2)	0.0120 (7)	
C26	0.6708 (4)	0.3553 (2)	1.2201 (2)	0.0125 (7)	
H26	0.5578	0.3389	1.2166	0.015*	
C27	0.7539 (4)	0.3816 (2)	1.1396 (2)	0.0097 (6)	
C28	0.6570 (4)	0.3854 (2)	1.0564 (2)	0.0100 (6)	
H28	0.5461	0.3742	1.0636	0.012*	
O1	1.0177 (3)	0.84419 (18)	0.90218 (16)	0.0114 (5)	
O2	0.6955 (3)	0.79453 (18)	0.82386 (17)	0.0129 (5)	
O3	0.9021 (3)	0.87169 (18)	1.09647 (16)	0.0120 (5)	
O4	0.5816 (3)	0.82436 (18)	1.01226 (16)	0.0128 (5)	
O5	1.1173 (3)	0.37770 (17)	0.86854 (16)	0.0107 (5)	
O6	0.7986 (3)	0.33961 (18)	0.79914 (16)	0.0118 (5)	
O7	1.0093 (3)	0.44105 (17)	1.07188 (16)	0.0090 (5)	
O8	0.7034 (3)	0.40163 (17)	0.97769 (16)	0.0116 (5)	
Cl1	1.36080 (9)	0.90539 (6)	0.87857 (6)	0.01455 (18)	
Cl2	1.19192 (11)	0.89319 (7)	0.51136 (6)	0.0199 (2)	
Cl3	1.11849 (9)	0.90686 (6)	1.26347 (6)	0.01406 (18)	
Cl4	0.58270 (11)	0.83281 (7)	1.45386 (6)	0.0221 (2)	
Cl5	1.45564 (9)	0.41888 (6)	0.83825 (6)	0.01241 (17)	
Cl6	1.27119 (11)	0.39485 (7)	0.47120 (6)	0.0197 (2)	
Cl7	1.20508 (9)	0.43012 (6)	1.23861 (6)	0.01256 (17)	
Cl8	0.64683 (11)	0.32313 (7)	1.40347 (6)	0.0193 (2)	
O9	0.8187 (3)	0.98658 (18)	0.94441 (17)	0.0133 (5)	
H9A	0.9028	1.0128	0.9186	0.016*	
H9B	0.8160	1.0363	0.9696	0.016*	
O10	0.7513 (3)	0.67389 (17)	0.97417 (17)	0.0118 (5)	
H10A	0.8109	0.6479	0.9410	0.014*	
H10B	0.7730	0.6619	1.0275	0.014*	
O11	0.8612 (3)	0.23683 (17)	0.95443 (17)	0.0120 (5)	
H11A	0.9147	0.2065	0.9205	0.014*	
H11B	0.8921	0.2170	1.0031	0.014*	

### Atomic displacement parameters (Å^2^)

**Table:**

	*U*^11^	*U*^22^	*U*^33^	*U*^12^	*U*^13^	*U*^23^
Zn1	0.00838 (19)	0.0114 (2)	0.0092 (2)	0.00159 (15)	−0.00065 (14)	0.00263 (14)
Zn2	0.00720 (19)	0.0103 (2)	0.00819 (19)	0.00178 (14)	−0.00008 (14)	0.00230 (14)
C1	0.0112 (16)	0.0069 (15)	0.0166 (17)	0.0020 (12)	0.0003 (13)	0.0044 (12)
C2	0.0133 (16)	0.0074 (15)	0.0109 (16)	0.0028 (12)	−0.0022 (13)	0.0019 (12)
C3	0.0106 (15)	0.0103 (16)	0.0142 (17)	0.0030 (13)	0.0036 (13)	0.0046 (12)
C4	0.0169 (17)	0.0127 (16)	0.0072 (15)	0.0031 (13)	0.0053 (13)	0.0025 (12)
C5	0.0153 (17)	0.0101 (16)	0.0164 (18)	0.0016 (13)	−0.0022 (14)	0.0021 (13)
C6	0.0109 (16)	0.0094 (16)	0.0104 (16)	−0.0005 (13)	0.0010 (12)	0.0019 (12)
C7	0.0117 (16)	0.0118 (16)	0.0123 (16)	0.0022 (13)	−0.0021 (13)	0.0021 (12)
C8	0.0136 (16)	0.0064 (15)	0.0076 (15)	0.0044 (12)	0.0010 (12)	0.0007 (11)
C9	0.0113 (16)	0.0074 (15)	0.0152 (17)	0.0033 (12)	0.0016 (13)	0.0029 (12)
C10	0.0176 (18)	0.0165 (18)	0.0106 (17)	0.0037 (14)	−0.0013 (13)	0.0011 (13)
C11	0.0158 (17)	0.0128 (17)	0.0137 (17)	0.0034 (14)	0.0057 (13)	0.0027 (13)
C12	0.0098 (16)	0.0130 (17)	0.0156 (17)	−0.0006 (13)	0.0025 (13)	0.0039 (13)
C13	0.0110 (16)	0.0089 (15)	0.0120 (16)	0.0029 (12)	−0.0001 (13)	0.0024 (12)
C14	0.0080 (15)	0.0112 (16)	0.0184 (18)	0.0001 (13)	−0.0010 (13)	0.0029 (13)
C15	0.0124 (16)	0.0087 (15)	0.0097 (16)	0.0018 (13)	−0.0003 (13)	0.0012 (12)
C16	0.0103 (15)	0.0084 (15)	0.0108 (16)	−0.0012 (12)	−0.0027 (12)	0.0009 (12)
C17	0.0084 (15)	0.0118 (16)	0.0134 (17)	0.0006 (13)	0.0002 (13)	−0.0007 (12)
C18	0.0176 (17)	0.0127 (16)	0.0075 (15)	0.0033 (13)	0.0047 (13)	0.0013 (12)
C19	0.0111 (16)	0.0173 (18)	0.0113 (16)	0.0044 (14)	−0.0025 (13)	0.0017 (13)
C20	0.0104 (15)	0.0113 (16)	0.0128 (16)	0.0051 (13)	0.0028 (13)	0.0025 (12)
C21	0.0097 (16)	0.0137 (17)	0.0138 (17)	0.0009 (13)	−0.0028 (13)	0.0015 (13)
C22	0.0107 (15)	0.0043 (14)	0.0090 (15)	−0.0026 (12)	0.0003 (12)	0.0017 (11)
C23	0.0091 (15)	0.0090 (15)	0.0111 (16)	−0.0005 (12)	−0.0031 (12)	0.0023 (12)
C24	0.0157 (17)	0.0088 (16)	0.0130 (17)	0.0030 (13)	−0.0010 (13)	0.0031 (12)
C25	0.0136 (16)	0.0101 (16)	0.0118 (16)	0.0007 (13)	0.0044 (13)	0.0052 (12)
C26	0.0092 (15)	0.0098 (16)	0.0174 (17)	0.0014 (13)	0.0033 (13)	−0.0003 (13)
C27	0.0103 (15)	0.0061 (15)	0.0103 (16)	−0.0009 (12)	−0.0013 (12)	−0.0006 (12)
C28	0.0057 (14)	0.0124 (16)	0.0100 (16)	0.0006 (12)	0.0007 (12)	−0.0014 (12)
O1	0.0089 (11)	0.0191 (13)	0.0080 (11)	0.0054 (9)	0.0018 (9)	0.0048 (9)
O2	0.0097 (11)	0.0143 (12)	0.0140 (12)	0.0018 (9)	0.0000 (9)	0.0032 (9)
O3	0.0105 (11)	0.0133 (12)	0.0108 (12)	0.0006 (9)	0.0001 (9)	0.0030 (9)
O4	0.0106 (11)	0.0154 (12)	0.0124 (12)	0.0039 (10)	−0.0012 (9)	0.0015 (9)
O5	0.0103 (11)	0.0127 (12)	0.0103 (11)	0.0039 (9)	0.0001 (9)	0.0045 (9)
O6	0.0068 (11)	0.0156 (12)	0.0123 (12)	0.0012 (9)	−0.0003 (9)	0.0036 (9)
O7	0.0069 (10)	0.0107 (11)	0.0083 (11)	0.0006 (9)	0.0000 (9)	0.0023 (9)
O8	0.0091 (11)	0.0133 (12)	0.0129 (12)	0.0038 (9)	−0.0004 (9)	0.0018 (9)
Cl1	0.0096 (4)	0.0199 (4)	0.0140 (4)	0.0040 (3)	−0.0021 (3)	0.0026 (3)
Cl2	0.0194 (4)	0.0286 (5)	0.0104 (4)	0.0033 (4)	0.0041 (3)	0.0053 (3)
Cl3	0.0105 (4)	0.0191 (4)	0.0124 (4)	0.0037 (3)	−0.0011 (3)	0.0028 (3)
Cl4	0.0225 (5)	0.0287 (5)	0.0146 (4)	0.0045 (4)	0.0097 (3)	0.0060 (4)
Cl5	0.0082 (4)	0.0159 (4)	0.0121 (4)	0.0018 (3)	−0.0015 (3)	0.0022 (3)
Cl6	0.0197 (4)	0.0279 (5)	0.0089 (4)	0.0024 (4)	0.0032 (3)	0.0020 (3)
Cl7	0.0089 (4)	0.0177 (4)	0.0105 (4)	0.0020 (3)	−0.0008 (3)	0.0043 (3)
Cl8	0.0196 (4)	0.0249 (5)	0.0147 (4)	0.0052 (4)	0.0092 (3)	0.0084 (3)
O9	0.0118 (11)	0.0118 (12)	0.0164 (13)	0.0028 (10)	0.0007 (9)	0.0038 (9)
O10	0.0104 (11)	0.0145 (12)	0.0112 (12)	0.0046 (9)	−0.0006 (9)	0.0026 (9)
O11	0.0118 (11)	0.0128 (12)	0.0133 (12)	0.0057 (9)	0.0012 (9)	0.0042 (9)

### Geometric parameters (Å, °)

**Table:**

Zn1—O1	2.040 (2)	C14—O4	1.229 (4)
Zn1—O2	2.096 (2)	C14—H14	0.9500
Zn1—O3	2.049 (2)	C15—O5	1.303 (4)
Zn1—O4	2.084 (2)	C15—C20	1.422 (4)
Zn1—O9	2.112 (2)	C15—C16	1.426 (5)
Zn1—O10	2.130 (2)	C16—C17	1.379 (5)
Zn2—O5	2.011 (2)	C16—Cl5	1.730 (3)
Zn2—O6	2.114 (2)	C17—C18	1.405 (4)
Zn2—O7	2.081 (2)	C17—H17	0.9500
Zn2—O7^i^	2.176 (2)	C18—C19	1.373 (5)
Zn2—O8	2.069 (2)	C18—Cl6	1.739 (3)
Zn2—O11	2.134 (2)	C19—C20	1.415 (5)
C1—O1	1.298 (4)	C19—H19	0.9500
C1—C2	1.422 (5)	C20—C21	1.452 (5)
C1—C6	1.429 (4)	C21—O6	1.226 (4)
C2—C3	1.378 (5)	C21—H21	0.9500
C2—Cl1	1.739 (3)	C22—O7	1.327 (4)
C3—C4	1.395 (5)	C22—C23	1.415 (4)
C3—H3	0.9500	C22—C27	1.427 (4)
C4—C5	1.374 (5)	C23—C24	1.378 (5)
C4—Cl2	1.741 (3)	C23—Cl7	1.737 (3)
C5—C6	1.407 (5)	C24—C25	1.404 (5)
C5—H5	0.9500	C24—H24	0.9500
C6—C7	1.454 (5)	C25—C26	1.370 (5)
C7—O2	1.225 (4)	C25—Cl8	1.736 (3)
C7—H7	0.9500	C26—C27	1.406 (5)
C8—O3	1.313 (4)	C26—H26	0.9500
C8—C9	1.419 (4)	C27—C28	1.453 (4)
C8—C13	1.431 (4)	C28—O8	1.228 (4)
C9—C10	1.376 (5)	C28—H28	0.9500
C9—Cl3	1.743 (3)	O7—Zn2^i^	2.176 (2)
C10—C11	1.393 (5)	O9—H9A	0.8400
C10—H10C	0.9500	O9—H9B	0.7416
C11—C12	1.379 (5)	O10—H10A	0.8400
C11—Cl4	1.743 (4)	O10—H10B	0.8234
C12—C13	1.403 (5)	O11—H11A	0.8400
C12—H12	0.9500	O11—H11B	0.8371
C13—C14	1.449 (4)		
			
O1—Zn1—O3	94.50 (9)	C12—C13—C14	115.3 (3)
O1—Zn1—O4	176.22 (8)	C8—C13—C14	123.4 (3)
O3—Zn1—O4	88.77 (9)	O4—C14—C13	128.2 (3)
O1—Zn1—O2	87.95 (9)	O4—C14—H14	115.9
O3—Zn1—O2	177.47 (10)	C13—C14—H14	115.9
O4—Zn1—O2	88.76 (9)	O5—C15—C20	124.4 (3)
O1—Zn1—O9	91.79 (10)	O5—C15—C16	120.1 (3)
O3—Zn1—O9	92.35 (9)	C20—C15—C16	115.5 (3)
O4—Zn1—O9	86.17 (9)	C17—C16—C15	123.2 (3)
O2—Zn1—O9	86.93 (9)	C17—C16—Cl5	119.1 (3)
O1—Zn1—O10	95.85 (9)	C15—C16—Cl5	117.7 (2)
O3—Zn1—O10	91.64 (9)	C16—C17—C18	119.2 (3)
O4—Zn1—O10	85.94 (9)	C16—C17—H17	120.4
O2—Zn1—O10	88.74 (9)	C18—C17—H17	120.4
O9—Zn1—O10	171.08 (9)	C19—C18—C17	120.4 (3)
O5—Zn2—O8	171.11 (9)	C19—C18—Cl6	120.6 (3)
O5—Zn2—O7	102.25 (9)	C17—C18—Cl6	119.0 (3)
O8—Zn2—O7	86.59 (9)	C18—C19—C20	120.3 (3)
O5—Zn2—O6	86.25 (9)	C18—C19—H19	119.8
O8—Zn2—O6	84.87 (9)	C20—C19—H19	119.8
O7—Zn2—O6	170.91 (9)	C19—C20—C15	121.2 (3)
O5—Zn2—O11	89.56 (9)	C19—C20—C21	116.7 (3)
O8—Zn2—O11	89.17 (9)	C15—C20—C21	122.1 (3)
O7—Zn2—O11	91.76 (9)	O6—C21—C20	127.1 (3)
O6—Zn2—O11	85.01 (9)	O6—C21—H21	116.4
O5—Zn2—O7^i^	93.02 (9)	C20—C21—H21	116.4
O8—Zn2—O7^i^	89.27 (9)	O7—C22—C23	120.8 (3)
O7—Zn2—O7^i^	81.32 (9)	O7—C22—C27	123.6 (3)
O6—Zn2—O7^i^	101.65 (8)	C23—C22—C27	115.6 (3)
O11—Zn2—O7^i^	172.99 (9)	C24—C23—C22	123.5 (3)
O1—C1—C2	120.8 (3)	C24—C23—Cl7	118.6 (2)
O1—C1—C6	124.5 (3)	C22—C23—Cl7	117.9 (3)
C2—C1—C6	114.7 (3)	C23—C24—C25	119.2 (3)
C3—C2—C1	123.8 (3)	C23—C24—H24	120.4
C3—C2—Cl1	118.4 (3)	C25—C24—H24	120.4
C1—C2—Cl1	117.7 (2)	C26—C25—C24	119.9 (3)
C2—C3—C4	119.3 (3)	C26—C25—Cl8	120.8 (3)
C2—C3—H3	120.4	C24—C25—Cl8	119.4 (3)
C4—C3—H3	120.4	C25—C26—C27	121.1 (3)
C5—C4—C3	120.1 (3)	C25—C26—H26	119.4
C5—C4—Cl2	120.9 (3)	C27—C26—H26	119.4
C3—C4—Cl2	119.0 (3)	C26—C27—C22	120.7 (3)
C4—C5—C6	120.7 (3)	C26—C27—C28	115.9 (3)
C4—C5—H5	119.7	C22—C27—C28	123.3 (3)
C6—C5—H5	119.7	O8—C28—C27	126.8 (3)
C5—C6—C1	121.4 (3)	O8—C28—H28	116.6
C5—C6—C7	115.7 (3)	C27—C28—H28	116.6
C1—C6—C7	122.9 (3)	C1—O1—Zn1	127.5 (2)
O2—C7—C6	128.1 (3)	C7—O2—Zn1	125.6 (2)
O2—C7—H7	116.0	C8—O3—Zn1	127.4 (2)
C6—C7—H7	116.0	C14—O4—Zn1	126.4 (2)
O3—C8—C9	120.5 (3)	C15—O5—Zn2	128.2 (2)
O3—C8—C13	124.5 (3)	C21—O6—Zn2	126.7 (2)
C9—C8—C13	115.0 (3)	C22—O7—Zn2	121.66 (19)
C10—C9—C8	123.7 (3)	C22—O7—Zn2^i^	115.7 (2)
C10—C9—Cl3	119.1 (3)	Zn2—O7—Zn2^i^	98.68 (9)
C8—C9—Cl3	117.2 (3)	C28—O8—Zn2	124.9 (2)
C9—C10—C11	119.2 (3)	Zn1—O9—H9A	109.5
C9—C10—H10C	120.4	Zn1—O9—H9B	144.2
C11—C10—H10C	120.4	H9A—O9—H9B	93.4
C12—C11—C10	120.3 (3)	Zn1—O10—H10A	109.5
C12—C11—Cl4	120.1 (3)	Zn1—O10—H10B	113.6
C10—C11—Cl4	119.6 (3)	H10A—O10—H10B	102.5
C11—C12—C13	120.4 (3)	Zn2—O11—H11A	109.5
C11—C12—H12	119.8	Zn2—O11—H11B	122.7
C13—C12—H12	119.8	H11A—O11—H11B	91.6
C12—C13—C8	121.3 (3)		
			
O1—C1—C2—C3	178.7 (3)	C23—C24—C25—Cl8	−178.5 (3)
C6—C1—C2—C3	−0.8 (5)	C24—C25—C26—C27	−1.7 (5)
O1—C1—C2—Cl1	−0.3 (4)	Cl8—C25—C26—C27	178.5 (3)
C6—C1—C2—Cl1	−179.8 (2)	C25—C26—C27—C22	−1.0 (5)
C1—C2—C3—C4	0.1 (5)	C25—C26—C27—C28	−177.6 (3)
Cl1—C2—C3—C4	179.0 (3)	O7—C22—C27—C26	−174.7 (3)
C2—C3—C4—C5	0.4 (5)	C23—C22—C27—C26	3.4 (5)
C2—C3—C4—Cl2	−179.4 (3)	O7—C22—C27—C28	1.7 (5)
C3—C4—C5—C6	−0.2 (5)	C23—C22—C27—C28	179.8 (3)
Cl2—C4—C5—C6	179.7 (3)	C26—C27—C28—O8	−176.2 (3)
C4—C5—C6—C1	−0.6 (5)	C22—C27—C28—O8	7.3 (5)
C4—C5—C6—C7	−178.9 (3)	C2—C1—O1—Zn1	165.8 (2)
O1—C1—C6—C5	−178.4 (3)	C6—C1—O1—Zn1	−14.8 (5)
C2—C1—C6—C5	1.0 (5)	O3—Zn1—O1—C1	−160.0 (3)
O1—C1—C6—C7	−0.3 (5)	O2—Zn1—O1—C1	19.3 (3)
C2—C1—C6—C7	179.2 (3)	O9—Zn1—O1—C1	−67.5 (3)
C5—C6—C7—O2	−179.0 (3)	O10—Zn1—O1—C1	107.9 (3)
C1—C6—C7—O2	2.7 (6)	C6—C7—O2—Zn1	9.7 (5)
O3—C8—C9—C10	−178.5 (3)	O1—Zn1—O2—C7	−16.7 (3)
C13—C8—C9—C10	2.1 (5)	O4—Zn1—O2—C7	161.5 (3)
O3—C8—C9—Cl3	−0.1 (4)	O9—Zn1—O2—C7	75.2 (3)
C13—C8—C9—Cl3	−179.5 (2)	O10—Zn1—O2—C7	−112.6 (3)
C8—C9—C10—C11	−0.7 (5)	C9—C8—O3—Zn1	170.2 (2)
Cl3—C9—C10—C11	−179.1 (3)	C13—C8—O3—Zn1	−10.4 (4)
C9—C10—C11—C12	−1.1 (5)	O1—Zn1—O3—C8	−169.0 (3)
C9—C10—C11—Cl4	177.4 (3)	O4—Zn1—O3—C8	12.9 (3)
C10—C11—C12—C13	1.4 (5)	O9—Zn1—O3—C8	99.0 (3)
Cl4—C11—C12—C13	−177.1 (3)	O10—Zn1—O3—C8	−73.0 (3)
C11—C12—C13—C8	0.2 (5)	C13—C14—O4—Zn1	4.1 (5)
C11—C12—C13—C14	179.1 (3)	O3—Zn1—O4—C14	−9.8 (3)
O3—C8—C13—C12	178.8 (3)	O2—Zn1—O4—C14	170.7 (3)
C9—C8—C13—C12	−1.8 (4)	O9—Zn1—O4—C14	−102.2 (3)
O3—C8—C13—C14	−0.1 (5)	O10—Zn1—O4—C14	81.9 (3)
C9—C8—C13—C14	179.3 (3)	C20—C15—O5—Zn2	−20.6 (5)
C12—C13—C14—O4	−175.5 (3)	C16—C15—O5—Zn2	160.1 (2)
C8—C13—C14—O4	3.4 (5)	O7—Zn2—O5—C15	−158.3 (3)
O5—C15—C16—C17	−178.4 (3)	O6—Zn2—O5—C15	24.9 (3)
C20—C15—C16—C17	2.2 (5)	O11—Zn2—O5—C15	110.0 (3)
O5—C15—C16—Cl5	0.7 (4)	O7^i^—Zn2—O5—C15	−76.5 (3)
C20—C15—C16—Cl5	−178.7 (2)	C20—C21—O6—Zn2	6.7 (5)
C15—C16—C17—C18	−1.1 (5)	O5—Zn2—O6—C21	−18.1 (3)
Cl5—C16—C17—C18	179.8 (3)	O8—Zn2—O6—C21	162.4 (3)
C16—C17—C18—C19	−1.5 (5)	O11—Zn2—O6—C21	−108.0 (3)
C16—C17—C18—Cl6	178.2 (3)	O7^i^—Zn2—O6—C21	74.2 (3)
C17—C18—C19—C20	2.8 (5)	C23—C22—O7—Zn2	151.4 (2)
Cl6—C18—C19—C20	−176.8 (3)	C27—C22—O7—Zn2	−30.6 (4)
C18—C19—C20—C15	−1.6 (5)	C23—C22—O7—Zn2^i^	−89.0 (3)
C18—C19—C20—C21	178.8 (3)	C27—C22—O7—Zn2^i^	89.0 (3)
O5—C15—C20—C19	179.8 (3)	O5—Zn2—O7—C22	−141.2 (2)
C16—C15—C20—C19	−0.8 (5)	O8—Zn2—O7—C22	37.8 (2)
O5—C15—C20—C21	−0.7 (5)	O11—Zn2—O7—C22	−51.2 (2)
C16—C15—C20—C21	178.7 (3)	O7^i^—Zn2—O7—C22	127.6 (3)
C19—C20—C21—O6	−172.9 (3)	O5—Zn2—O7—Zn2^i^	91.22 (10)
C15—C20—C21—O6	7.6 (5)	O8—Zn2—O7—Zn2^i^	−89.78 (9)
O7—C22—C23—C24	174.7 (3)	O11—Zn2—O7—Zn2^i^	−178.85 (9)
C27—C22—C23—C24	−3.4 (5)	O7^i^—Zn2—O7—Zn2^i^	0.0
O7—C22—C23—Cl7	−2.5 (4)	C27—C28—O8—Zn2	15.4 (5)
C27—C22—C23—Cl7	179.4 (2)	O7—Zn2—O8—C28	−31.0 (3)
C22—C23—C24—C25	1.0 (5)	O6—Zn2—O8—C28	145.8 (3)
Cl7—C23—C24—C25	178.1 (3)	O11—Zn2—O8—C28	60.8 (3)
C23—C24—C25—C26	1.7 (5)	O7^i^—Zn2—O8—C28	−112.4 (3)

Symmetry codes: (i) −*x*+2, −*y*+1, −*z*+2.

### Hydrogen-bond geometry (Å, °)

**Table:**

*D*—H···*A*	*D*—H	H···*A*	*D*···*A*	*D*—H···*A*
O9—H9A···O3^ii^	0.84	2.02	2.790 (3)	153
O9—H9B···O1^ii^	0.74	2.45	3.015 (3)	134
O10—H10A···O7^i^	0.84	2.24	2.998 (3)	150
O10—H10B···O5^i^	0.82	1.95	2.741 (3)	161
O11—H11A···O3^i^	0.84	2.17	2.931 (3)	151
O11—H11B···O1^i^	0.84	1.93	2.751 (3)	169

Symmetry codes: (ii) −*x*+2, −*y*+2, −*z*+2; (i) −*x*+2, −*y*+1, −*z*+2.
